# Incidental Discovery of Complications of Cryptorchidism During Laparoscopic Inguinal Hernia Surgery

**DOI:** 10.1155/cris/9852275

**Published:** 2025-03-10

**Authors:** Keita Sato, Natsuki Hashiba, Koji Takahashi, Hirotaka Shibuya

**Affiliations:** Department of Surgery, Ise Red Cross Hospital, Ise City, Mie, Japan

## Abstract

**Background:** Cryptorchidism is one of the most common congenital anomalies in newborn males, with the majority diagnosed in infancy and treated surgically before puberty. In some cases, cryptorchid testes are discovered incidentally during inguinal hernia repair in adults, requiring appropriate management at the time of surgery.

**Case Presentation:** Case 1: A 58-year-old male underwent emergency laparoscopic surgery for a left incarcerated hernia. Intraoperatively, an M2 indirect inguinal hernia with omental strangulation of the spermatic cord was identified. Despite attempts to pull the testis down manually, it did not descend into the scrotum, leading to the diagnosis of cryptorchidism. The spermatic cord was excised and a 3D mesh was placed to cover the hernia defect. Case 2: A 33-year-old man with Noonan syndrome presented with localized pain and swelling in the right groin. Laparoscopy revealed an indirect L3 inguinal hernia and incidentally an intra-abdominal testis was found. The testis and spermatic vessels were found to terminate intra-abdominally, confirming the diagnosis of cryptorchidism. An orchiopexy was performed to secure the testis subcutaneously in the scrotum and the hernia was repaired with a 3D mesh.

**Conclusion:** The safety of mesh-based hernia repair in cases of cryptorchidism with concomitant inguinal hernia has been previously reported. In cases where preoperative palpation is difficult due to pain, intraoperative traction of the testis may help differentiate between cryptorchidism and retractile testis. In addition, orchiopexy may serve as a temporary measure, with consideration of staged orchiectomy if necessary.

## 1. Introduction

Cryptorchidism, or undescended testis are among the most prevalent congenital anomalies in male infants, with a typical diagnosis and management during early childhood. The conventional treatment for pediatric cases involves orchidopexy, which is a surgical procedure that relocates the testis into the scrotum. However, in adults, the necessity for surgical intervention is often driven by the risk of torsion, malignancy, and fertility complications. In inguinal hernia surgery, preoperative intraperitoneal computed tomography is relatively uncommon, since the decision on the indication for surgery is based primarily on physical examination findings. However, it has been reported that approximately 90% of patients with undescended testes have an inguinal hernia, including subclinical ones [1]. The frequency of patients with inguinal hernias complicated by undescended testes is reported to be approximately 6%–7% [2]. Therefore, it is possible that an undescended testicle may be discovered incidentally during surgery even if a planned radical hernia repair is performed. In these case reports, we present a successful case of laparoscopic surgery for an adult inguinal hernia complicated by cryptorchidism.

## 2. Case Presentation

### 2.1. Case 1

**Figure 1:**
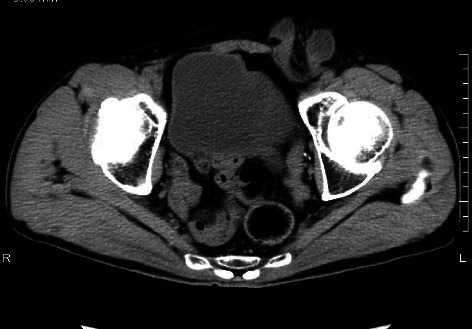
CT findings at the time of presentation (Case 1: intestinal incarceration in the left inguinal region).

A 58-year-old male patient was transferred to our hospital via ambulance, complaining of persistent abdominal and left inguinal pain. Physical examination revealed that the abdomen was slightly distended, and the left groin was tender. No abnormalities were observed in the right inguinal region or testis. A CT scan of the abdomen was performed (Figure 1), which revealed a small intestinal insert into the inguinal canal. However, no assessment of the testicular location was made at that time. The diagnosis of a hernia incarceration was made, and after performing a manual repair, emergency laparoscopic surgery was chosen.

**Figure 2:**
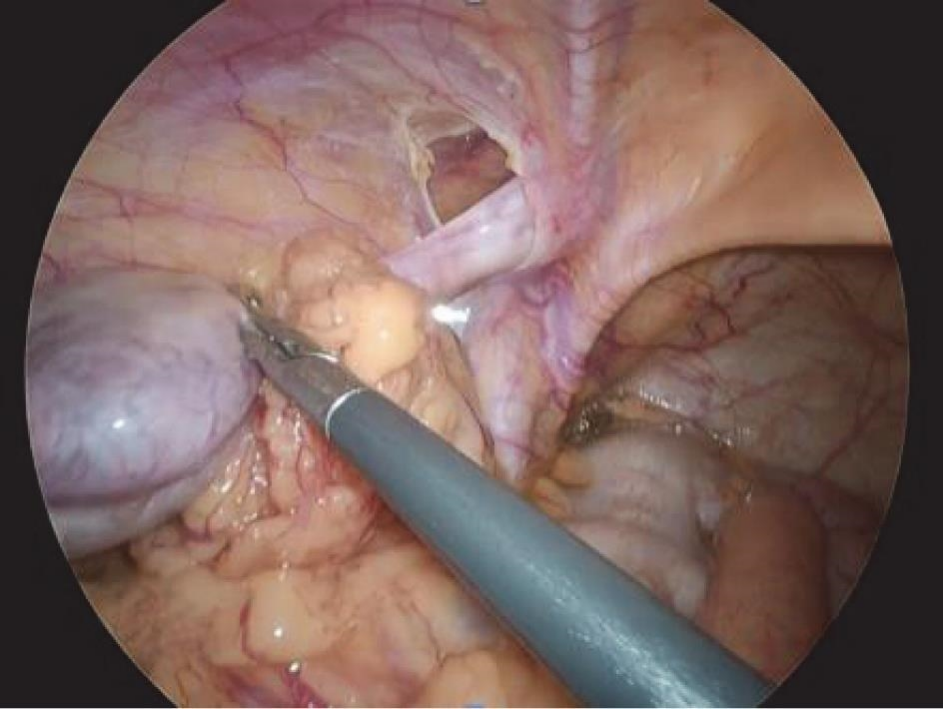
Intraoperative findings (Case 1). Left indirect hernia was observed, and the testis and spermatic cord confined within the inguinal canal were diagnosed as intracanalicular type.

A left-sided indirect hernia (M2) was identified, and the greater omentum was entrapped within the sac. The right inguinal region had no hernia findings and the testes were normal. When the large mesh was returned to the peritoneal cavity, the atrophied testis was easily elevated into the abdominal cavity (Figure 2). Intraoperatively, the testis was tracted from outside the body, but it did not descend within the scrotum and was diagnosed as undescended testis. Due to its poor appearance, the spermatic cord was excised and removed using an automated suture device. Myopectineal orifice (MPO) was subsequently covered with 3D mesh (size L) and fixed with tacking. The histopathological examination of the testis revealed no findings suggestive of malignancy.

### 2.2. Case 2

A 33-year-old male patient diagnosed with Noonan's syndrome was admitted to the hospital with complaints of severe and localized pain and swelling in the right inguinal region. Palpation of the scrotum revealed an absence of testicular tissue, but a detailed palpation of the testis could not be performed due to the pain. Imaging studies showed incarcerated hernia of greater omentum and emergency surgery was performed.

**Figure 3:**
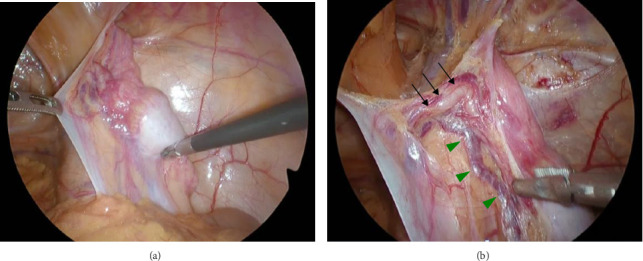
Intraoperative findings (Case 2). (a) Right indirect hernia was identified. The testis remaining in the abdominal cavity was observed, and communication between the gonadal vessels and vas deferens was noted. (b) Dissection revealed that the whitish mass was a testis and that it was in traffic with the vas deferens (arrow) and testicular vessels (arrow head).

Laparoscopy revealed a 6–7 cm whitish mass in the right inguinal region covered by an L3 indirect hernia and peritoneum (Figure 3a). The left inguinal region showed no hernia findings and the testes were normal. The mass was atrophic and located on the ventral side of the inguinal canal, communicating with the vas deferens and spermatic vessels (Figure 3b). A diagnosis of cryptorchidism was made. As there was no preoperative informed consent for the excision of the testis, orchidopexy (fixation of the testis) was performed. The testis was fixed in the subcutaneous tissue of the scrotum, and the MPO was closed with a 3D mesh (size M) and fixed with tacking. In this case, the patient was followed up with palpation of the testicular area every few months after surgery and with ultrasound or CT scan every 6 months. If there are palpable indurations or changes in size on imaging studies, we will recommend removal of the testes.

## 3. Discussion

In Case 1, a detailed physical examination was not possible due to pain caused by incarceration. However, intraoperative findings revealed that after resection of the omentum, the scrotum immediately returned to a high inguinal position, suggesting incomplete descending testis. Based on this observation, the diagnosis of cryptorchidism was considered appropriate. In addition, testicular ischemia was suspected in this case, justifying orchiectomy.

In contrast, in Case 2, a retractile testis was also considered in the differential diagnosis. However, the presence of a congenital condition (Noonan syndrome) and the termination of the testicular vessels and vas deferens proximal to the internal inguinal ring (Figure 3b) supported the diagnosis of cryptorchidism.

When evaluating a testis in an abnormal position, it is important to distinguish between cryptorchidism and retractile testis. Embryologically, the differentiation is a function of whether the descent of the testis is complete or not. However, palpation alone may not be sufficient to differentiate high position cryptorchidism from a retractile testis. In adults, unlike children, the size of the scrotum is usually adequate for palpation, which may facilitate preoperative diagnosis. However, as seen in the present case, severe pain may preclude thorough examination, necessitating reliance on intraoperative findings. If the testis can be retracted into the scrotum by gentle traction during surgery, it is likely to be a retractile testis; otherwise, cryptorchidism is more likely.

As indicated in this report, the pivotal consideration in adult cases is the necessity of testicular removal. The prognosis of retractile testis is generally favorable, with normal development and no significant impact on future fertility [3]. However, some studies comparing the histological findings of retractile and normal testes have reported abnormalities in retractile testis [4]. While preservation is typically acceptable, orchidopexy might be worth considering. For cryptorchidism, the necessity of orchiectomy is well-established and unquestionable [5]. Also, orchiectomy is a relative indication when orchidopexy cannot be attempted, for example, because traction to the testis is not possible.

We identified a total of 11 reports (71 cases) of laparoscopic repair for adult inguinal hernia with cryptorchidism including our case using the keywords “inguinal hernia,” “adult,” “laparoscopic surgery,” “cryptorchidism,” or “undescended testis” based on PubMed search for all English articles [6–16] (Table 1).

The 71 cases ranged in age from 12 to 68, with a majority discovered between 20 and 30 years of age. Cryptorchidism types were classified based on localization into intraabdominal type and intracanalicular type. The intraabdominal type was predominantly observed.

Historically, surgical intervention for cryptorchidism has often included orchiectomy (Table 1). Although laparoscopic orchidopexy has also been performed, including in our cases, this approach may reflect the lack of informed consent specifically addressing orchiectomy and the perception that the risk of malignancy does not sufficiently justify testicular removal. Notably, no studies have reported complications such as infection, suggesting that simultaneous hernia repair with prosthetic materials and undescended testis surgery is feasible.

In adults, prolonged intra-abdominal presence of the testis increases the likelihood of infertility [17]. Therefore, careful consideration of informed consent and the risk of tumor development is critical when choosing a surgical approach. Although orchiectomy is generally preferred, in cases where testis preservation is chosen, techniques such as orchidopexy, as observed in Case 2, could be considered. These methods allow for easy follow-up and facilitate possible secondary removal if needed. In our cases, we also have a policy of recommending that the patient undergo a testis removal if there are abnormal findings or changes in size with palpation and imaging every few months postoperatively.

## 4. Conclusions

The differentiation between cryptorchidism and retractile testis requires careful evaluation and intraoperative palpation should be incorporated to achieve an accurate diagnosis. When cryptorchidism is diagnosed or suspected, laparoscopy may help in confirming the diagnosis. Laparoscopic hernia repair and orchiectomy at the same time is a feasible and safe approach. Furthermore, orchidopexy is a valuable option to facilitate staged testicular removal if such a procedure becomes necessary in the future.

## Figures and Tables

**Table 1:** Reported cases of adult inguinal hernia with cryptorchidism treated by laparoscopic surgery.

No.	Year	Age	Side/hernia	Type of inguinal hernia	Operation of hernia	Side/cryptochidism	Type of cryptochidism	Operation of cryptochidism	Complication	Reference
1	1994	25, 61 (2 cases)	Right	Indirect	TAPP	Right	Intra-abdoimnal	Orchiectomy	None	[1]
2	1995	22	Right	Indirect	TAPP	Right	Intra-abdoimnal	Orchiectomy	NS	[2]
3	2002	30	Bilateral	Indirect	TAPP	Bilateral	Intra-abdoimnal	Right orchidopexy Left orchiectomy	None	[3]
4	2003	20–27 (44 cases) average: 23	Various	Various	TAPP	Various	Intra-abdoimnal 35 Intracanalicular 7	Orchiectomy	2 Pneumoscrotum	[4]
5	2004	12–28 (4 cases)	Right1, left3	Indirect	TAPP	Various	Intra-abdoimnal	Orchiectomy	NS	[5]
6	2007	26−38 (11 cases)	Various	Various	TAPP	Various	Various	Various	None	[6]
7	2008	35	Right	Indirect	TAPP	Left	Intra-abdoimnal	Orchiectomy	None	[7]
8	2014	22	Left	Indirect	TEP	Right	Intracanalicular	Orchiectomy	None	[8]
9	2015	49, 38 (2 cases)	Right	Indirect	TEP	Right	Intracanalicular	Orchiectomy	None	[9]
10	2017	53	Right	Indirect	TAPP	Right	Intra-abdoimnal	Orchidopexy	None	[10]
11	2020	68	Right	Indirect	TAPP	Right	Intracanalicular	Orchiectomy	None	[11]
12	2024 (Our cases)	33, 58	Right/left	Indirect	TAPP	Right/left	Intra-abdoimnal/ intracanalicular	Orchidopexy/ orchiectomy	None	–

## Data Availability

Data sharing is not applicable to this article as no datasets were generated or analyzed during the current study.
